# *Plectranthus amboinicus* (Lour.) Spreng: Botanical, Phytochemical, Pharmacological and Nutritional Significance 

**DOI:** 10.3390/molecules21040369

**Published:** 2016-03-30

**Authors:** Greetha Arumugam, Mallappa Kumara Swamy, Uma Rani Sinniah

**Affiliations:** Department of Crop Science, Faculty of Agriculture, Universiti Putra Malaysia, Serdang, Selangor, Darul Ehsan 43400, Malaysia; greetha82@yahoo.co.uk

**Keywords:** *Plectranthus amboinicus*, Indian borage, pharmacology, biological activities, nutrition, phytochemistry, botany, medicinal plant

## Abstract

*Plectranthus amboinicus* (Lour.) Spreng. is a perennial herb belonging to the family Lamiaceae which occurs naturally throughout the tropics and warm regions of Africa, Asia and Australia. This herb has therapeutic and nutritional properties attributed to its natural phytochemical compounds which are highly valued in the pharmaceutical industry. Besides, it has horticultural properties due to its aromatic nature and essential oil producing capability. It is widely used in folk medicine to treat conditions like cold, asthma, constipation, headache, cough, fever and skin diseases. The leaves of the plant are often eaten raw or used as flavoring agents, or incorporated as ingredients in the preparation of traditional food. The literature survey revealed the occurrence 76 volatiles and 30 non-volatile compounds belonging to different classes of phytochemicals such as monoterpenoids, diterpenoids, triterpenoids, sesquiterpenoids, phenolics, flavonoids, esters, alcohols and aldehydes. Studies have cited numerous pharmacological properties including antimicrobial, antiinflammatory, antitumor, wound healing, anti-epileptic, larvicidal, antioxidant and analgesic activities. Also, it has been found to be effective against respiratory, cardiovascular, oral, skin, digestive and urinary diseases. Yet, scientific validation of many other traditional uses would be appreciated, mainly to discover and authenticate novel bioactive compounds from this herb. This review article provides comprehensive information on the botany, phytochemistry, pharmacology and nutritional importance of *P. amboinicus* essential oil and its various solvent extracts. This article allows researchers to further explore the further potential of this multi-utility herb for various biomedical applications.

## 1. Introduction 

At present, plant-based medicines are widely employed in various public health practices throughout the globe as they are safe and cost-effective, and efficiently combat various deadly diseases and help in maintaining good health [1]. Herbal medicines are very commonly used in Unani, Ayurveda, Sidda, folk and other traditional practices of healthcare management [2]. According to the estimation by the World Health Organization, about 80% of people on the globe are still dependent on traditional herb-based medications due to their low cost, easy accessibility and likely negligible side effects in comparison to allopathic medicines [2,3,4]. Certainly, many of the leading active drug molecules of plants and their derivatives used presently in allopathic medicine are mainly due to the understanding of traditional medical practices for curing diseases [2]. Modern drug discovery research is governed by natural plant-based compounds and their products, followed by synthetic chemical drugs. Currently, natural products are considered as a major source of medicaments and, hence, they are extensively used by pharmaceutical industries. This has led towards increased global demand for medicinal plants in the modern era of natural medicine, leading to exploration and exploitation of new plant sources for their medicinal properties [3,5,6]. The Lamiaceae members of plant species belonging to commercially important genera, such as *Plectranthus*, *Salvia, Ocimum* and *Mentha*, are attributed with a rich diversity of ethnobotanical benefits. More than 300 species of *Plectranthus* are reported all over the tropical and warm regions of the old world, including Asia, Africa and Australia [7]. In over 85% of the literature, documentation of *Plectranthus* is on the therapeutic values of this genus followed by its nutritional and horticultural properties attributed to its aromatic nature and essential oil producing capability [8,9]. *Plectranthus amboinicus* (Loureiro) Sprengel is one of the most documented species in the family Lamiaceae. *P. amboinicus*, also commonly known as Indian borage, and is a fleshy, succulent herb famous for its distinct oregano-like flavor and odor. It is one of the most cited species in the Lamiaceae family, especially for its medicinal properties, accounting for 68% of all customary applications of this genus [10]. This herb is widely used by indigenous people of tropical rain forests, either in folk medicine or for culinary purposes. This is mainly due to its natural production of an essential oil with high amounts of bioactive compounds such as Carvacrol [11], Thymol [12] β-Caryophyllene, α-Humulene, γ-Terpinene, *p*-Cymene, α-Terpineol and β-Selinene, identified in the oil component of its leaves [13,14]. These biochemical components exhibit various biological properties [15,16] and are widely used in folk medicine to treat conditions like cold, asthma, constipation, headache, cough, fever and skin diseases. The leaves of the plant are often eaten raw or used as flavoring agents, or incorporated as ingredients in the preparation of traditional food. The chopped leaves are also used as a substitute for sage (*Salvia officinalis*) in meat stuffing [17]. With this background, the present review was undertaken to present complete facts on the multifarious medical benefits of *P. amboicus*. This review is a compiled survey of information on various aspects of *P. amboinicus* including botany, distribution, wild relatives, phytochemistry, medicinal and nutritional properties and other benefits. The available information was retrieved from many search engines including ScienceDirect, Google Scholar, PubMed, Scopus and SciFinder *etc*.

## 2. Botanical Description

### 2.1. Taxonomy

*P. amboinicus* (Loureiro) Sprengel is a member of the family, Lamiaceae. or mint family. The paleotropical oil-rich genus, *Plectranthus* belongs to the subfamily Nepetoideae. It comprises about 300 species of annual or perennial herbs or subshrubs which are often succulents [18]. Many species of *Plectranthus* have economical and medicinal values. Among them, *P. amboinicus* is one the most important aromatic medicinal succulent plants that possess distinctive smelling leaves with short soft erect hairs.

### 2.2. Morphological Feautures

*P. amboinicus* is a succulent shrub with a tendency for climbing or creeping. It can reach over 1 m in height and even more in width in the wild [18,19,20]*.* This sprawling large succulent herb is fleshy and highly aromatic. The fleshy stems grow about 30–90 cm, either with long rigid hairs (hispidly villous), or tomentose (densely covered with soft, short and erect hairs, pubescent) [18,19]. Leaves are undivided (simple), broadly ovate to suborbicular with a tapering tip (ovate) and very thick; they are pubescent (thickly studded with hairs), with the lower surface possessing the most numerous glandular hairs, giving a frosted appearance [20]. The taste of this leaf is pleasantly aromatic with an agreeable and refreshing odor. Flowers are on a short stem (shortly pedicelled), pale purplish in dense whorls at distant intervals in a long slender raceme. Flowers have a bell shaped calyx and the throat is smooth inside with two lips, the upper lip being ovate and thin, the lower lip having four narrow teeth. The corolla is pale purplish and five times longer than the calyx, with a short tube, inflated throat and short lips [19,20]. Fruit nutlets are smooth, pale brown in color, 0.7 mm long and 0.5 mm wide. *P. amboinicus* rarely flowers and seeds are difficult to collect [19].

### 2.3. Origin, Wild Relatives and Geographical Distribution

The name *Plectranthus* derives from the Greek words “plectron”, meaning spur, and “Anthos”, meaning flower, in reference to the spur-shaped flowers of some members of the genus [21]. Due to lack of precise morphological features to distinguish species within the genus *Plectranthus* and its closely associated genera, numerous taxonomic problems with the naming of species have resulted in misplacement of species in some closely linked genera such as *Coleus, Solenostemon* and *Englerastrum* [10]. The species *P. amboinicus* was originally classified under the genus *Coleus* but was moved to the *Plectranthus* genus, although both names are sometimes seen in the literature today. This species also has the greatest number of synonyms (Table 1). The synonyms of *P. amboinicus* include *P. aromaticus* Roxb., *Coleus aromaticus* Benth. and *C. amboinicus* Lour. [10]. *P. amboinicus* is of unknown origin, but is possibly from Africa and India [18], and it has since been distributed and cultivated pantropically. The type specimen of the species was collected in Amboina, Moluccas, resulting in its species name *amboinicus* [22]. Taxonomic revisions of *Plectranthus* have taken place on a regional rather than international basis and thus have contributed to a misunderstanding of the same species [10]. The traditional uses around the world are represented in Table 1.

### 2.4. Cultivation

*P. amboinicus* is a fast-growing plant usually propagated by stem cuttings. This preferred propagation through vegetative means is because it rarely seeds or sets seed [23]. The herb grows easily in a well-drained, semi-shaded location. It is found to grow well under tropical and subtropical locations. It was also found to adapt well in cooler climates if grown in a pot and brought indoors, or moved to a warm, sheltered position during winter [19]. The plant should be watered only sparingly. *P. amboinicus* grows best in rich, compost soil with neutral pH and high humidity, but if there is excess water in the ground its roots might start to rot. On the other hand, it copes well with severe droughts, as it has lots of water stored in its succulent flesh. It also survives well with severe heat and scorching sun, as well as strong shade, but grows best under partial shade. For all those reasons, it is very easy to grow indoors and that is why it is becoming more and more popular as a house plant in northern Europe*. P. amboinicus* cannot withstand temperatures lower than 0 °C and is stressed even when it is colder than 10 °C. In general, very meager information is available on the methods/practices used for commercial cultivation and harvesting of this herb.

## 3. Phytochemistry

A large and growing body of literature has investigated the chemical composition and pharmacological properties of *P. amboinicus*. The literature survey has emphasized the occurrence of different classes of phytocompounds including 76 volatiles and 30 non-volatile compounds. More recently, interest among phytochemists and biologists has focused on the isolation of specific bioactive compounds of *P. amboinicus* and comprehending their pharmacological importance. However, the chemical profile and the accumulation pattern of bioactive constituents in different parts of the plant and their essential oil content varies depending on various parameters, such as geographical features, climate and different stages of plant material collection [2]. Moreover, the method of extraction and identification can also lead to variations in phytochemical composition. Presently, only a few reports have stated the isolation and authentication of individual compounds of *P. amboinicus.* Therefore, correct identification, isolation and quantification of phytocompounds is very much necessary in order to understand their pharmacological and biological significance. *P. amboinicus* is reported to contain several classes of phytochemicals including monoterepenoids, diterpenoids, triterpenoids, sesquiterpenoids, phenolics, flavonoids and esters. The details of these bioactive constituents are discussed in this section.

### 3.1. Volatile Composition of P. amboinicus

**Figure 1:**
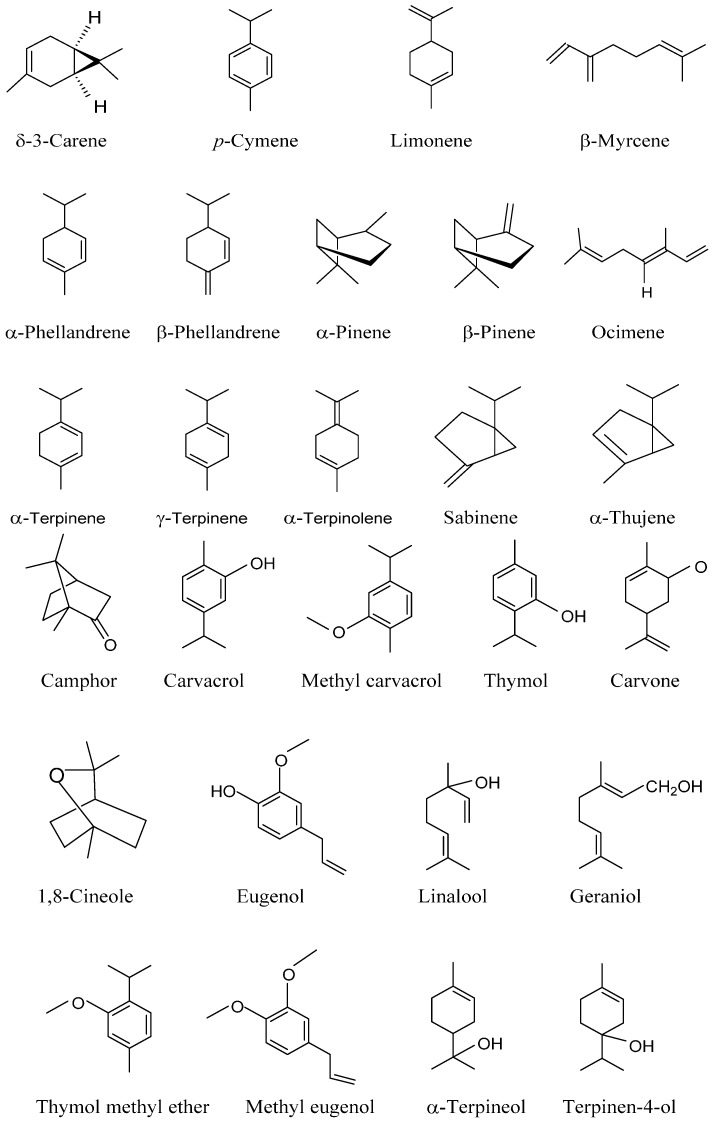

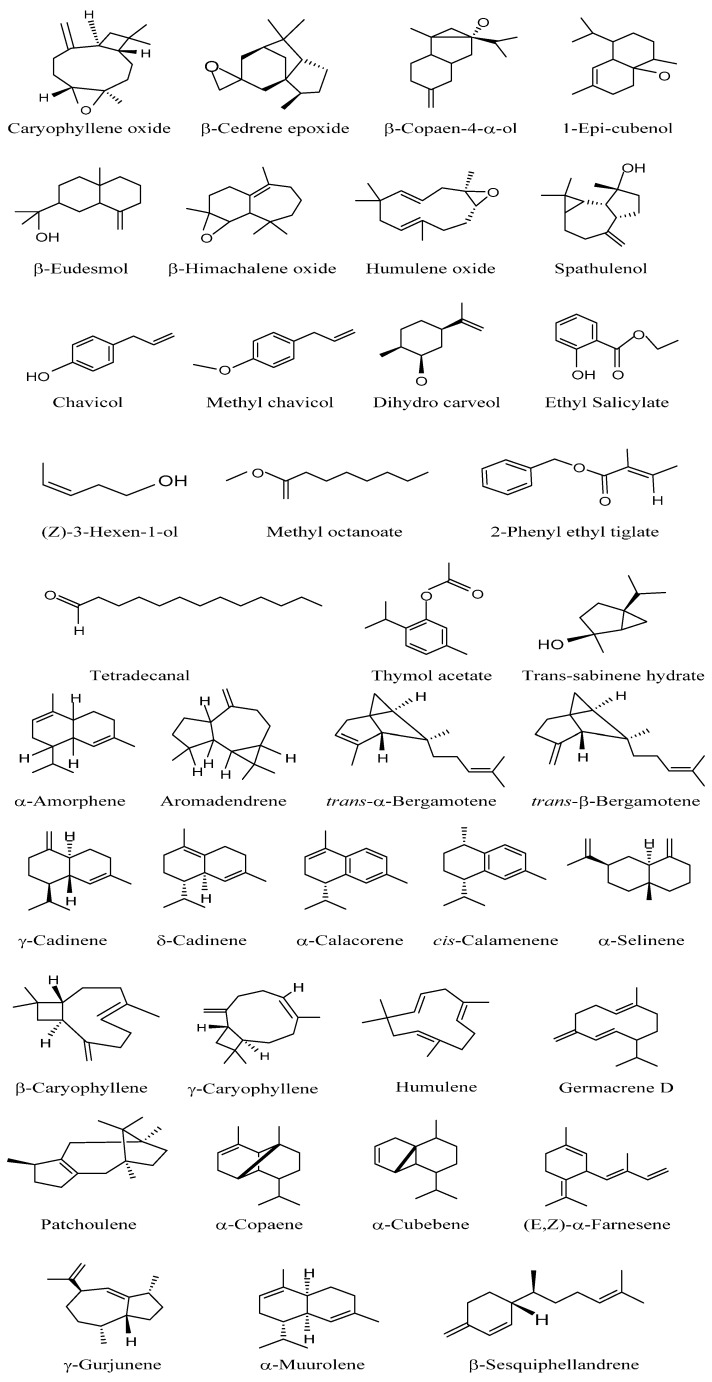
The structures of some of the volatile chemical constituents.

The essential oil obtained from the leaves and stem explants was shown to contain a total of 76 volatile constituents. The essential oil contained a copious quantity of the two major phenolic compounds, namely, carvacrol and thymol, which are pharmaceutically appreciated for various culinary properties. The quality as well as quantity of chemical compounds occurring in the essential oil is directly related to its biological functions. *P. amboinicus* oil is rich in oxygenated monoterpenes, monoterpene hydrocarbons, sesquiterpene hydrocarbons and oxygenated sesquiterpenes [10]. The leaf essential oil of *P. amboinicus* is particularly rich in phenolic monoterpenes such as Thymol and Carvacrol, which are speculated to exert various pharmacological properties [10,17,20,24]. Table 2 represents the details of these volatile compounds, while Figure 1 depicts the structural details of some of these major compounds.

A hydro distillation method using a Clevenger type apparatus for 3–4 h is commonly employed for extracting *P. amboinicus* essential oil. However, volatile constituents of *P. amboinicus* leaf obtained by using hexane extraction, steam distillation and supercritical CO_2_ extraction methods were shown to have a chemical difference [25]. The hexane extraction method produced the highest oil yield (6.52%) compared to steam distillation (0.55%) and supercritical CO_2_ extraction methods (1.40%). It was also noticed that there was a difference in aroma of the extracts which was attributed to qualitative and quantitative dissimilarity in chemical composition. In another study, a solid phase micro extraction (SPME) method showed the presence of eucalyptol in *P. amboinicus* leaves [17,26]. The volatile constituents of *P. amboinicus* leaves collected from Uganda were extracted with head space solid phase microextraction (HS-SPME) and their analysis using the gas chromatography–mass spectroscopy (GC-MS) electron impact ionization method revealed the presence of Linanol (50.3%) as the major component [27,28]. The other volatiles observed were Carvacrol (10.3%), Geranyl acetate (11.75), Nerol acetate (11.6%), γ-Terpinene (3.2%), *p*-Cymene (2.9%), Nerol (2.3%), α-4-Carene (1.3%), Caryophyllene (1.2%) and β-Myrcene (0.8%).

The chemical constituents of *P. amboinicus* essential oil differed with the collected samples from diverse geographical places. In India, *P. amboinicus* essential oil was reported to possess volatiles such as Carvacrol (43.1%), Thymol (7.2%), Eugenol (6.4%), Chavicol (5.3%) and Et-salicylate (3.2%) [40], which varied from the constituents observed by Baslas and Kumar [41] with Thymol (41.30%), Carvacrol (13.25%), 1,8-Cineole (5.45%), Eugenol (4.40%) and β-Caryophyllene (4.20%). Likewise, analysis of essential oil obtained from wild growing *P. amboinicus* plants in Bangalore, India showed the presence of 36 compounds [29]. The major compounds identified were Carvacrol (53%–67%), *p*-Cymene (6.5%–12.6%), β-Caryophyllene (7.4%), Caryophyllene oxide (2.2%) and α-Terpinene (5.9%–15.5%). For the first time, there was an occurrence of the compounds eugenol and methyl eugenol in Indian (Andra Pradesh) *P. amboinicus* oil [12].

GC and GC-MS techniques have indicated the occurrence of Thymol (94.3%), followed by Carvacrol (1.2%), 1,8-Cineole (0.8%), *p*-Cymene (0.3%), Spathulenol (0.2%) and Terpinen-4-ol (0.2%) as the major constituents of Indian *P. amboinicus* leaf essential oil. Investigation of volatiles of *P. amboinicus* collected from Mysore, Karnataka, India showed the existence of Carvacrol (70%), β-Caryophyllene (6.2%), *p*-Cymene (5.6%) and α-Terpinolene (5.3%) as the main components [13]. The volatile composition of aerial parts and flowers of *P. ambonicus* growing in Belgaum, Western Ghats region of North West Karnataka, India have been studied by Joshi *et al.* [33] using gas chromatography outfitted with a flame ionization detector (GC-FID) and GC-MS. The results revealed the occurrence of 12 components from aerial parts oil and 4 constituents from flower oil and represented 94.29% and 90.25% of the total oil, respectively. In both the essential oils, the major compound observed was Carvacrol (50.98% in flowers and 77.16% in aerial parts oils). They also noticed the chemotype nature of the plants with minor quantities of α-Calacorene, Methyl chavicol and α-Corocalene from aerial parts oil. However, *P. amboinicus* collected from Western Ghats (Siruvani hills), Tamilnadu, India contained only 14% of Carvocrol. Other chief constituents observed were Thymol (18%), *cis*-Caryophyllene (18%) and *p*-Cymene (10.83%) [34]. Yet, the presence of chemical variation in the leaf essential oils had been evident from the plants collected from the same state [42,44]. A total of 10 volatile compounds were identified with dominant constituents as Carvacrol (50.7%), β-caryophyllene (13.1%) and patchoulane (8.7%) [42], while the occurrence of Carvacrol was not evident from the study of Uma *et al.* [44]. They reported the occurrence of 3-Methyl-4-isopropyl phenol (31.70%) as the major constituent followed by Squalene (10.07%), Caryophyllene (2.36%) and Phytol (8.44%). Similarly, the essential oil of *P. amboinicus* leaves obtained from the Cuddalore district of Tamilnadu, India was shown to contain a total of 26 compounds, with Carvacrol (28.65%) and Thymol (21.66%) observed as the major compounds [14]. Some of the other major components included α-Humulene (9.67%), γ-Terpinene (7.76%), Undecanal (8.29%), *p*-Cymene (6.46%), α-Terpineol (3.28%), β-Selinene (2.01%) and Caryophyllene oxide (5.85%). 

Likewise, plants growing in the same locality were shown to contain 26 compounds and among them Carvacrol (29.25%), Thymol (21.66%), Undecanal (8.29%) and α-Humulene (9.67%) were the major compounds [35]. About 28 volatile compounds were revealed from the aerial parts of *P. amboinicus* cultivated in Uttarakhand, India by using GC and GC-MS analysis. Among the 16 identified compounds, Thymol (83.39%) was the major component. The other important components observed were 1-Octen-3-ol, Caryophyllene oxide, Terpine-4-ol, *trans*-Caryophyllene, Eugenol and α-Cadinol [39]. Likewise, chemical investigation of essential oil of *P. amboinicus* collected from Himalayan foothill and mid-hill regions of India were reported by Verma *et al.* [47]. They identified 44 constituents representing 86%–99% of the oil composition using GC and GC-MS analysis. Some of the major components observed were Thymol (57.7%–66.4%), γ-Terpinene (5.8%–11.7%), *p*-Cymene (4.1%–14.2%), (*E*)-Caryophyllene (2.6%–3.6%), Caryophyllene oxide (1.3%–1.5%) and 1-Octen-3-ol (1.0%–1.8%). Essential oil of *P. amboinicus* from Mauritius was characterized by Gurib-Fakim *et al.* [30] using capillary GC and GC-MS analysis and the results revealed Carvacrol (41.3%) and Camphor (39%) as the dominant volatiles.

The leaf essential oil obtained from Martinique, France was found to contain Carvacrol (72%) as a chief phenolic component along with newly identified compounds such as (*Z*)-1,3-Hexadiene (0.1%), (*E*,*Z*)-α-Farnesene (0.2%), (*Z*)-3-Hexenol (0.6%), α-Muurolene (0.2%) and (*E*,*E*)-α-Farnesene (0.2%) [46]. *P. amboinicus* oil from Cambodia was shown to contain largely Thymol (57.4%). Other major constituents such as Carvacrol (13.5%), *p*-Cymene (5.2%) and γ-Terpinene (5.6%) were also noticed [36]. Likewise, 64.3% of Thymol was identified from Brazilian *P. amboinicus* leaf essential oil [37]. Other major compounds identified were *p*-Cymene (10.3%), β-Caryophyllene (2.8%) and γ-Terpinene (9.9%). Liquid-solid chromatography (LSC), Gas-liquid chromatography (GLC) and GC-MS analysis of *P. amboinicus* plants from Cuba revealed Carvacrol (64%) as the major component among the identified 13 terpene hydrocarbons and seven oxygenated compounds [43]. Similarly, Velasco *et al.* [38] identified 15 volatile constituents in the essential oil of *P. amboinicus* collected from Venezuela and the major component observed was Carvacrol (65.2%). Capillary GC and GC-MS analysis of *P. amboinicus* essential oil from the Archipelago of Comoros was carried out by Hassani *et al.* [31] and the results showed the existence of Carvacrol (23.0%), Camphor (22.2%), δ-3-Carene (15.0%), γ-Terpinene (8.4%), *p*-Cymene (7.7%) and α-Terpinene (4.8%) as the major constituents of the oil. The essential oil from Uganda consisted of Linalool (50.3%), Carvacrol (14.3%), Nerol acetate (11.6%) and Geranyl acetate (11.7%) as the major components as revealed by GC-MS analysis [40]. Essential oil of *P. amboinicus* leaves from Serdang, Malaysia was shown to contain Carvacrol (19.29%), 3-Carene (20.78%) and Camphor (17.96%) as the major volatile constituents [32].

The essential oil yield and its main chemical constituents are also influenced by the environmental factors and different seasons. Mallavarupu *et al.* [29] have revealed that the quality of essential oil will be superior when collected during September. The oil content was found to be higher in the plants harvested during September in comparison to the plants harvested during May. Moreover, the compositions also differed among the two harvest times. About 68% of oxygenated monoterpenes, 11% of sesquiterpenes and 3.3% oxygenated sesquiterpenes were found in the oil distilled from the September harvest time, whereas, the oil obtained from the May harvest comprised higher amounts of monoterpenes (35.7%). The major compounds such as Carvacrol (67.0%), β-Caryophyllene (7.4%), α-Humulene (2.1%) and Caryophyllene oxide (2.2%) were found to be higher in oil obtained during September, while the oil distilled during May showed the presence of *p*-Cymene (12.6%), γ-Terpinene (15.5%), Carvacrol (53.0%) and β-Caryophyllene (4.3%). A GC-MS study was carried out by Roja *et al.* [45] to identify the chemical constituents of *P. amboinicus* oil from the leaves of the tissue culture plants, *in vitro* root cultures as well as parent plants. The results revealed the presence of similar volatile constituents, though the parent plants and root cultures contained 21 compounds in comparison to only 15 compounds noticed in the tissue culture plants. The concentration of Thymol compound was found to be 0.012, 0.29 and 0.009% (fresh weight) in the tissue culture plants, *in vitro* roots and the parent plants, respectively. They also suggested that thymol and *cis*-Caryophyllene can be produced from the tissue culture plants as oil yield and Thymol concentration was found to be similar to the parent plants. Interestingly, the root cultures had a three times higher Thymol content after a period of four weeks of day growth. This suggested the possible use of root cultures for producing Thymol and *cis*-Caryophyllene on a large scale.

### 3.2. Non-Volatile Chemical Constituents of P. amboinicus

**Figure 2:**
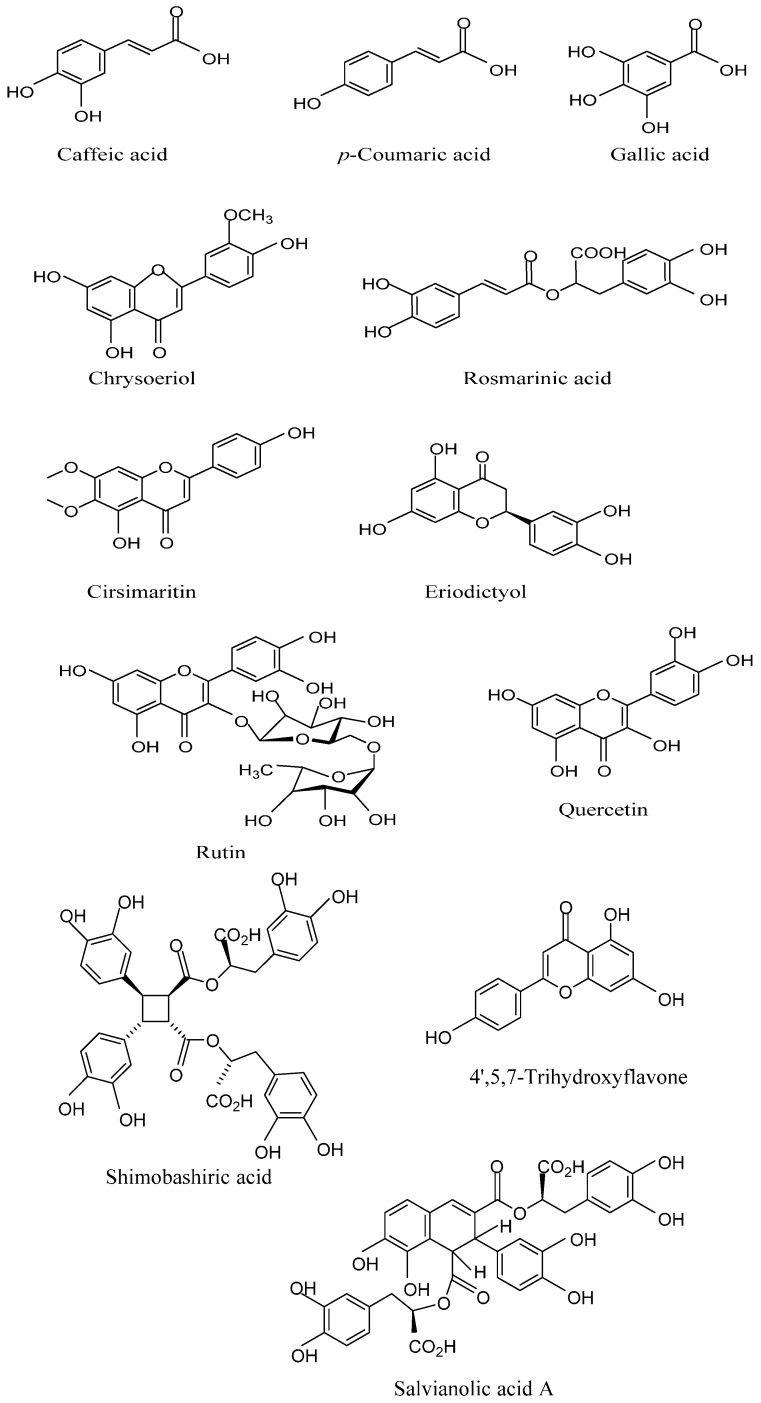
The structures of some of the non-volatile chemical constituents.

A total of 30 non-volatile constituents have been identified from *P. amboinicus* according to our literature survey [17,48]. These non-volatile chemical components included phenolic acids, flavonoids, monoterpene hydrocarbons, sesquiterpene hydrocarbons, oxygenated monoterpenes and esters. The details of these phytocompounds are exemplified in Table 3 and some of the important and major compound structures are illustrated in Figure 2. The chloroform extract of *P. amboinicus* air dried leaves was subjected to fractionation using the silica gel column chromatography technique to separate non-volatile compounds [49]. Later, the isolated compounds were identified by using ultra violet (UV), 1-D nuclear magnetic resonance (NMR) and 2-D NMR spectroscopy as three flavones, namely, Cirsimaritin, Salvigenin and Chrysoeriol. High pressure liquid chromatography (HPLC) analysis was carried out to analyze the major compound, Carvacrol, present in the aqueous extract of *P. amboinicus* plants obtained from Taichung of Taiwan [50]. The results showed an abundance of Carvacrol occurrence in the aqueous extract, with 1.88 mg/g of the extract. Eight compounds from the ethyl acetate fraction of *P. amboinicus* leaves collected from Egypt were isolated and identified [48]. Based on the UV, NMR spectra and their physical data, these isolated compounds were identified as 5,4′-Dihydroxy-3,7- dimethoxy flavone (3-methoxy genkwanin), 5,4′-Dihydroxy-6,7-dimethoxy flavone (Crisimaritin), *p*-Coumaric acid (Hydroxy cinnamic acid), Caffeic acid, 3,5,7,3′,4′-Pentahydroxy flavanone (Taxifolin), Rosmarinic acid, Apigenin and 5-*O*-Methyl-luteolin. For the first time, they reported the occurrence of 3-Methoxy genkwanin, *p*-Coumaric acid and 5*-O*-Methyl-luteolin in this plant. In the same study, total phenolic content was higher in the stem extract (9.6 mg/g) compared to the leaf extracts (8.4 mg/g) and the root extracts (5.4 mg/g).

The tannin content was found to be highest in the root extracts (126 μg/g), followed by the leaf (90 µg/g) and stem extracts (81 µg/g). The use of high resolution Ultra Performance Liquid Chromatography (UPLC)-MS analysis identified the presence of Rosmarinic acid, Chrysoeriol, Caffeic acid and *p*-Coumaric acid in the ethyl acetate fractions of stems and roots. However, the compounds, Eriodyctiol, Luteolin and Quercetin were found only in the ethyl acetate fractions of stems [48]. Bhatt *et al.* [51] have analyzed the phytochemicals present in the methanolic stem extracts of *P. amboinicus*. Their study revealed the existence of total phenolic content (49.9 mg gallic acid equivalent (GAE)/g extract), condensed tannins (0.7 mg tannic acid equivalent (TAE)/g extract) and total flavonoids (26.6 mg rutin equivalent (RE)/g extract). Further, HPLC analysis of the extract confirmed the occurrence of bioactive polyphenols such as Rosmarinic acid (6.16 mg/g extract), Rutin (0.32 mg/g extract), Caffeic acid (0.77 mg/g extract), Gallic acid (0.26 mg/g extract), *p*-Coumaric acid (0.10 mg/g extract) and Quercetin (0.15 mg/g extract). The chemical composition of aqueous leaf extracts of *P. amboinicus* was reported to contain tannins, flavonoids, saponins, polyuronides and steroid glycosides [28]. In the same study, about 11 phytocompounds constituting 97.6% of the total extract were confirmed using GC-MS analysis. The major components included Linalool (50.3%), Carvacrol (14.3%), Geranyl acetate (11.7%) and Nerol acetate (11.6%). Chen *et al.* [52] have isolated and identified four bioactive compounds with transcription factor inhibition activity from aerial parts (stem and leaves) of *P. ambonicus* collected from Thailand. They separated the compounds from water extracts using HPLC column-based fractionation to detect the compounds using mass spectrometry and NMR analysis. The identified non-volatiles included Rosmarinic acid, Thymoquinone, Shimobashiric acid and Salvianolic acid.

## 4. Bioactivities of *P. amboinicus*

### 4.1. Antimicrobial Activities

*P. amboinicus* extract, from crude extract to an essential oil, contains innumerable biological constituents owing to its chemical diversity. Plant phytochemicals possess antimicrobial activity against a wide range of bacteria, yeast and mold, but vary in quantity and quality depending on the bioactive constituents [3,4,53]. Its wide range of chemical diversity containing phytochemicals such as terpenes, alcohols, acetones, phenols, acids, aldehydes and esters is often used as components in the pharmaceutical industry. Table 4 provides the detailed antimicrobial activities of different parts of *P. amboinicus* and its major compounds. 

#### 4.1.1. Antibacterial Activities

Bacteria are prokaryotic microorganisms usually found on the surface of the skin, mucosal layer and intestinal tract of humans and animals. The genus *Staphylococcus* is one example of common bacteria found to reside on the skin and in mucous membrane and is mostly harmless. Yet, there are dangerous bacteria classified as human pathogens, causing contagious diseases with a fatal prognosis [54]. These bacteria are usually inhibited by taking antibiotics. However, recently drug resistance developed by microbes is increasingly observed and is a global phenomenon. Therefore, continuous exploration of medicinal plants for effective drugs is an ongoing process. From early years, *P. amboinicus* has been used as folk medicine to fight pathogenic bacterial activity. In Cuba, a decoction of the leaves was given to patients suffering from chronic cough or tuberculosis and later scientific studies revealed *P. amboinicus* having anti-*Mycobacterium tuberculosis* activity [55]. Hot water extract of *P. amboinicus* leaves inhibited growth of pathogens, *Escherichia coli* and *Salmonella typhimurium* while stimulating the growth of *Lactobacillus plantarum* [56]. This antibacterial activity of plant extracts is most likely due to the combined effect of adsorption of polyphenols to bacterial membranes with membrane disruption and subsequent leakage of cellular contents [57,58], and the generation of hydro peroxide from polyphenols [59]. Further, it was shown that unsterilized ethanolic leaf extract of *P. amboinicus* exhibits antibacterial activity against diabetic wound pathogens, *E. coli*, *S. aureus*, *P. mirabilis*, *P. aeruginosa* and *K. pneumonia* [60]. Current attention is focused on inhibition of antibiotic-resistant bacteria as it is gradually becoming a major problem in the medical industry. Essential oil of *P. amboinicus* is reported to have a synergistic effect on the antibiotic toxicity toward resistant bacterial strains when combined with aminoglycosides. This makes *P. amboinicus* essential oil a possible source of a natural product with bacterial resistance-modifying activity [61]. In another study, Vijayakumar *et al.* [62] used leaf extract of *P. amboinicus* to biologically synthesize zinc oxide nanoparticles (Pam-ZnO NPs). These Pam-ZnO NPs successfully controlled the growth of methicillin-resistant *Staphylococcus aureus* biofilms (MRSA ATCC 33591) at the concentration of 8–10 g·mL^−1^.

#### 4.1.2. Antifungal Activities

There is also vast evidence that *P. amboinicus* plays a crucial role in hindering the growth of disease causing fungus. However, little is known of the derivatives and its effectiveness when used together with industrial drugs. In evaluating the interference of *P. amboinicus* essential oil on the anti-*Candida* activity of some clinically used antifungals (itraconazole, ketoconazole and amphotericin B), it showed a diverse level of interference. Essential oil exhibited prominent interference on the activity of itraconazole, providing a synergic effect on *C. albicans*, *C. tropicalis*, *C. krusei* and *C. stellatoidea.* Whereas, interference on the anti-yeast activity of ketoconazole was antagonic and synergic when interacting with *C. albicans, C. guilliermondii* and *C. stellatoidea.* Amphotericin B, on the other hand, showed a small interference on the anti-yeast activity [63]. In another research, antifungal activity of the volatile oil was studied against various fungi by an agar well diffusion susceptibility test. In that, growth of *Aspergillus ochraceus*, *Aspergillus niger* and *Penicillium* sp. was inhibited by 60%, 64% and 60%, respectively, with 10 µL of volatile oil [13].

#### 4.1.3. Antiviral Activities

A large number of active agents are available for the symptomatic treatment of sexually transmitted diseases (STDs) and acquired immune deficiency syndrome (AIDS). Nevertheless, the emergence of drug-resistant strains and dose-limiting toxic effects has complicated the treatment of these diseases and necessitated the search for new antimicrobial substances from various sources. In the last decade, major advancements have been reported in the field of “microbicides”, *i.e.*, compounds or formulations which, when applied topically can prevent the transmission of STDs including AIDS [28]. These include a few from plant sources such as gossypol derivatives, praneem polyherbal preparations and plantibodies. Likewise, extracts of *P. amboinicus* were tested and reported to have antiviral activity against Herpes Simplex Virus-1 (HSV1) [64] and anti-HIV inhibition activity [65]. Besides that, ethanolic extract of *P. amboinicus* was reported to have selective antiviral activity on Vero cell lines at 0.1 mg/mL minimum inhibitory concentration when tested against HSV1 and Vesicular Stomatitis (VSV) viruses [66]. 

### 4.2. Respiratory Disorders

*P. amboinicus* is frequently cited in the treatment of chronic coughs, asthma, bronchitis and sore throat in India and the Caribbean Islands [67,68,69]. In accordance with that, leaves of *P. amboinicus* had positive bronchodilator activity when tested on guinea pigs [70]. In Eastern Cuba, essential oil from aerial parts of *P. amboinicus* is used to treat asthma [11]. Decoction or juice made from leaves together with other herbs is also taken orally to control asthma. This decoction is also used to treat catarrhal infections where it clears the excessive build-up of thick phlegm or mucus in an airway or cavity of the body [71]. Collectively, the reason behind this could be high amounts of Carvacrol [11] and Thymol [12] found in the essential oil of the plant. Carvacrol and Thymol are an excellent expectorant and used to treat various respiratory disorders. It is suggested that a drink or a bath of *P. amboinicus* juice/decoction can be a worthy treatment for influenza, cough, bronchitis and throat problems [72].

### 4.3. Activity against Digestive Diseases

*P. amboinicus* is a popular treatment for dyspepsia, indigestion and diarrhea, and a carminative in India and Africa [30,67,68,77]. In India, the leaves of *P. amboinicus* are consumed along with buttermilk, yogurt, or any other probiotic sources during pathogen-induced diarrhea. The leaves are known to have a prebiotic effect on the probiotic bacteria *Lactobacillus plantarum*. They utilize the phytoconstituents of the leaves by producing necessary metabolic enzymes. A detailed examination by Shuba and Bhatt [56], describes the mode of hot water extract (HWE) of *P. amboinicus* leaves on growth inhibition of *Escherichia coli* and *Salmonella typhimurium* (pathogens) while stimulating the growth of *Lactobacillus plantarum.* Sodium dodecyl sulfate polyacrylamide gel electrophoresis (SDS-PAGE) gel showed the presence of phenolic acid decarboxylase enzyme induced in the presence of HWE, which indicated the utilization of polyphenols by the bacteria. Cells grown on HWE also showed β-galactosidase activity, indicating their ability to utilize sugars present in HWE. This provides evidence in the traditional use of the leaves in the alleviation of diarrhea by accelerating microbial gut balance during infection. In addition, *P. amboinicus* juice obtained from pounded leaves is used as a drink to cure constipation in Indonesia and Malaysia [77].

### 4.4. Antiepileptic Activity

Various literatures have reported the use of *P. amboinicus* in the treatment of nervous disorders, including epilepsy and convulsions [68]. In Cuba, it is used as an anticonvulsive and antiepileptic drug [11]. Bhattacharjee and Manjumder [94], tested the anticonvulsant activity of the leaf, stem and root alcoholic extract separately on Swiss albino mouse models by maximal electric shock-induced seizures and pentylenetetrazole-induced seizures. They found significant anticonvulsant activity in both the models with alcoholic leaf extract recording the highest activity. They also predicted that the presence of alkaloids, flavonoids and saponins in these extracts may be responsible for this activity.

### 4.5. Antitumorigenic Activities

The antitumor activity of hexane extracts of *P. amboinicus* has been reported [78]. The results showed a significant inhibition on the growth of Sarcoma-180 tumor in mice treated with the hexane extracts of *P. amboinicus*. A dose of 350 mg/kg of hexane extracts of *P. amboinicus* significantly reduced the growth of S-180 tumor with 66% inhibition, while doses of 100, 150 and 250 mg/kg reduced the inhibition to 44%, 45% and 47%, respectively. There were no significant differences in body weight before and after the treatments. This was comparable to metrotexat, a cancer-treating drug which may cause very serious, life-threatening side effects but reduces 100% of tumoral growth. Nevertheless, hexane extracts of *P. amboinicus* is plant-based, therefore, the severity of side effects can be reduced while the tumoral growth is being destroyed. *P. amboinicus* ethanolic extract showed significant anticancer activity through inducing apoptosis in the A549 (human lung cancer) cell line [79].

### 4.6. Anti-Inflammatory Activities

The hexane extract (HE) of *P. amboinicus* was also shown to exhibit anti-inflammatory activity [78]. A significant reduction of the paw edema was observed at doses of 150, 250 and 350 mg/kg of the HE of *P. amboinicus*. The highest percentages of reduction of the paw edema were observed in the groups treated with 250 (41%) and 350 mg/kg (33%) of the HE of *P. amboinicus*. Interestingly, the lowest percentage of inhibition of paw edema was observed in the groups that were treated with 10 mg/kg of the indomethacin, a non-steroidal antiinflammatory drug. Rheumatoid arthritis is a chronic inflammatory disease. The activator protein-1 (AP-1) controls the expression of inflammatory cytokines, whereas tumor necrosis factor (TNF-α) plays a key role in the pathogenesis of inflammatory bone resorption. The active constituents of *P. amboinicus* were shown to possess AP-1 and TNF-α inhibitory activities [52]. However, they further suggested validating the AP-1 and TNF-α inhibitory potential of *P. amboinicus* for the treatment of rheumatoid arthritis. Treatment of leaf methanolic extracts of *P. amboinicus* resulted with moderate to high anti-inflammatory activity in experimental mice [34]. *In vitro* and *in vivo* studies have revealed the potent anti-inflammatory activity of aqueous extract of *P. amboinicus* [50]. The anti-inflammatory activity was related to modulation of antioxidative enzymes in the liver with a decreased malondialdehyde level. Also, they observed the production of TNF-α and cyclooxygenase 2 (COX-2) in the tissue of edema paw induced in mice. *In vitro* studies revealed the production of proinflammatory mediators in RAW 264.7 cells. Most recently, Silitonga *et al.* [80] reported the significant improvement of immunoglobulin levels (IgG, IgM) and lysozyme activity in rats when treated with ethanolic leaf extract of *P. amboinicus.*

### 4.7. Wound Healing Activities

Few studies have investigated the ability of *P. amboinicus* to reduce blood sugar levels. Some of the phytochemicals found in *P. amboinicus* have been proven to play an important role towards blood sugar level lowering mechanisms (Table 4). This herb has the ability to prevent or decrease the risk of infection and its complications in diabetic patients [84]. Application of a paste prepared using *P. amboinicus* showed an enhanced wound healing ability by immune-stimulation in diseased giant murrels [85]. Likewise, *P. amboinicus* leaves and root derived paste (10%) has been shown to exhibit thorough epithelialization on the excision wound in albino rats after 12 days of application [86]. The use of polyherbal suspension prepared from *P*. *amboinicus* and *Punica granatum* was shown to exhibit good wound healing properties in laboratory mice [87]. Further, ethanolic extract of *P*. *amboinicus* reduced the wound area by up to 76.6% in diabetic mice induced by monosodium glutamate. It was observed that the plant extract promoted wound healing by increased wound contraction, enhancing collagen deposition and reducing the wound epithelialization period [60].

### 4.8. Effects against Skin Diseases

*P. amboini*cus has been used in Brazil since the early days for the treatment of skin ulcerations caused by *Leishmania braziliensis* [89]. In India, the juice of the leaves is used to treat skin allergies [90]. It is also used to treat burns in Asian regions [68]. When the leaf paste is baked on a flame and applied to cuts or burns, it acts as an antiseptic and promotes healing [91]. Essential oil of *P. amboinicus* also inhibits the growth of dandruff-causing fungus *Malassezia furfur*, and was tested using the agar diffusion method and compared against Ketaconazole-based shampoo as the standard [92].

### 4.9. Effects against Animal and Insect Bites

Leaves of *P. amboinicus* are also used as a poultice for centipede and scorpion bites in Asian regions, including Malaysia [68]. It is reported that aqueous extracts (0.706 mg/mL and 0.406 mg/mL) of *P. amboinicus* to be more than 70% efficient when tested against fibroblast cell lysis [93]. This implies the aqueous extracts to have a tendency to be scorpion (*Heterometrus laoticus*) venom antidotes. However, the same paper also reported its cytotoxicity to be questionable.

### 4.10. Lactogenic Activity

In Indonesia, *P. amboinicus* is used as a traditional food in soup to stimulate lactation for the month or so following childbirth. The leaves are commonly consumed by mothers who have given birth in North Sumatra, in particular the Batak tribe. The leaves of this herb are believed to increase the production of breast milk due to the high content of nutrients, especially iron and carotene. Consumption of leaves significantly increases minerals such as iron, potassium, zinc and magnesium in milk, thus, improving the infant’s weight and health holistically [80].

### 4.11. Antioxidant Activities

The essential oil *P. amboinicus* possesses a significant antioxidant property against stress-created in cell line-induced lung cancer in both (*in vitro* and *in vivo*) models which could be due to the presence of phytochemical compounds such as Carvocrol and Thymol. Non-enzymatic antioxidant-reduced glutathione was found to be increased in the *P. amboinicus* essential oil treated mice. The presence of important bioactive compounds confirms the possibility of its use in pharmaceutical drug formulations. The use of essential oil of *P. amboinicus* is cheaper than natural drug formulation and also without any side effects on the animal model reported [34]. For the first time, the aqueous leaf extract of *P. amboinicus* was reported to possess higher superoxide- scavenging, nitric oxide-scavenging and ferrous ion-chelating capacity [99]. A report by Bhatt and Negi [16] showed the highest polyphenolic content with appreciable total antioxidant and 1,1-diphenyl-2-picrylhydrazyl (DPPH) free radical-scavenging properties in the solvent extract of *P. amboinicus* leaves. Similarly, Khanum *et al.* [100] found lower content of total flavonoids and total phenolics and antioxidant activity in an ethanolic leaf extract of *P. amboinicus.*

### 4.12. Oral Diseases

Caries and periodontal disease are especially of concern to public health, where they affect a large part of the population. *P. amboinicus*, rich in carvacrol, has shown an antagonistic effect when used with mouthwash to avoid bacterial growth in the oral cavity [76]. This could be a potential alternative treatment for diseases related to oral cavities. 

### 4.13. Larvicidal Potential

Mosquitoes have the ability of carrying and transmitting human and animal diseases across countries causing hundreds of millions of clinical cases and millions of deaths annually. Senthilkumar and Venkatesalu [14] reported the possible use of *P. amboinicus* essential oil as a low cost eco-friendly resource for inhibiting the malarial vector mosquito population. The LC_50_ values of the oil were found to be 33.5 and 28.3 ppm after 12 and 24 h, respectively. Likewise, Lima *et al.* [73] reported larvicidal activity (LC_50_ value: 58.9 ± 0.4 μg/mL) of the essential oil of *P. ambonicus* against the mosquito (*Aedes aegypti*) which is a chief vector of dengue, yellow fever and dengue hemorrhagic fever. In another study, the essential oil of *P. amboinicus* was shown to act as a good larvicidal agent against the mosquito, *Anopheles gambiae* after 48 h [47]. In an investigation by Baranitharan *et al.* [74], the highest larvicidal activity against *Aedes aegypti, Anopheles stephensi* and *Culex quinquefasciatus* was found in the ethyl acetate leaf extracts of *P. amboinicus*. More recently, Jayaraman *et al.* [75] have reported the larvicidal potential of different solvent extracts of *P. amboinicus* leaves against *Aedes aegypti*, *Culex quinquefasciatus*, and *Anopheles stephensi. P. amboinicus* zinc oxide nanoparticles (Pam-ZnO NPs) showed 100% mortality of fourth instar mosquito larvae of *Anopheles stephensi, Culex quinquefasciatus* and *Culex tritaeniorhynchus* at the concentration of 8 and 10 g/mL. The histopathological studies of Pam-ZnO NPs-treated *A. stephensi* and *C. quinquefasciatus* larvae revealed the presence of damaged cells and tissues in the mid-gut. The damaged tissues suffered major changes, including rupture and disintegration of the epithelial layer and cellular vacuolization [62]. This biological control could be slow, but a long-lasting, inexpensive alternative and harmless to the ecosystem.

### 4.14. Activity against Cardiovascular Disorders

*P. amboinicus* is also used in the Caribbean, to treat congestive heart failure [67]. The aqueous extracts of the fresh leaves of *P. amboinicus* exhibited dose-dependent positive inotropic activity in the isolated frog heart without affecting the heart rate [88]. This may be attributed to the increase in sodium influx thereby causing greater intracellular availability of calcium. In this report the bioactivity of the tissue-cultured extracts of *P. amboinicus* to the parent plant was also described. Both extracts from tissue-cultured and parent plant produced a comparable significant effect indicating that they both can be used as a source of biochemical production. 

### 4.15. Activity against Genitourinary Diseases 

The leaves of *P. amboinicus* are frequently utilized in the treatment of urinary diseases in the Amazon and India [69,95]. This species is also reported to relieve kidney troubles and treat vaginal discharges, and is taken as a drink after childbirth [67]. Urolithiasis is a condition when stony concretions form in the bladder or urinary tract. Many remedies have been employed during the treatment of urinary stones. The juice of *P. amboinicus* has been used as a natural remedy to dilute the crystals in the urinary tract in India from ancient times [96]. The antilithiotic activity of the concentrated fresh juice of the leaves of *P. amboinicus* is proved by Jose *et al.* [97]*.* The said study on urine analysis revealed significant reduction in calcium, oxalates and total protein level compared to the control. Further histopathological results showed an absence of crystal and normal-sized tubules with a single epithelial lining. He suggested this antilithiotic activity could be associated with calcium oxalate origin. The diuretic properties of ethanolic and aqueous extracts of *P. amboinicus* were evaluated by determination of urine volume and electrolyte concentration in male albino rats. Furosemide (10 mg/kg) was used as a standard, while normal saline (0.9%) was used as a control. Both ethanolic and aqueous extracts (500 mg/kg) have shown a significant increase in the volume of urine and urinary concentration of Na, K and Cl ions and were comparable to furosemide. This study concludes that the leaves of *P. amboinicus* possess diuretic activities [98].

### 4.16. Analgesic Activity 

In Africa, *P. amboinicus* is used as a remedy for headaches [81]. The aqueous extract of *P. amboinicus* leaves showed an analgesic and anti-inflammatory property, mainly modulated by controlling inhibition of proinflammatory mediators [52]. It is also used to treat musculo-skeletal conditions such as a stiff neck and backache [82,83]. 

### 4.17. Activity against Other Diseases

*P. amboinicus* is an important herb in Asia and South America for the treatment of infectious diseases such as fevers [67,101], cholera and meningitis [102]. It also used to treat sensory disorders associated with ear and eye problems. For example, *P. amboinicus* seed oil is a treatment for acute edematous otitis acuta in Polynesia [103], whereas in India its leaves are rubbed into the eyes to alleviate conjunctivitis [67].

## 5. Culinary Uses

### 5.1. Nutritional Values

Herbs have been used extensively in culinary purposes since ancient times. Many delicious cuisines we enjoy contain various dietary herbs to increase the taste and flavor of the food. Herbal plants also have lots of health benefits attributed to their nutritional content [17,27]. Hence, *P.* *aromaticus* can be a good source of nutritive compounds which help to enhance the taste and also prolong the shelf life of food products. A study validates the presence of high minerals, precisely calcium and potassium, at 0.158% and 0.138%, respectively [17]. The detailed nutritional components are presented in Table 5 [17,19,27]. These minerals are necessary to build and maintain strong bones and to retain normal function of heart, kidneys, muscles and nerves. *P. amboinicus* also has a significant content of iron at 0.262%. Iron is an important component of hemoglobin aids red blood cells to carry oxygen throughout the body. Hemoglobin represents about two-thirds of the body’s iron and its deficiency causes anemia. Adding to that, this plant also contains total Xanthophylls (0.356 mg/g of dry weight of the plant) which consist of Neoxanthin, Violaxanthin, Leutin, Zeaxanthinics. It also has α-Carotene (0.157 mg/g of dry weight) and β-Carotene (0.0035 mg/g of dry weight) [104]. All this makes *P. amboinicus* a unique dietary supplement.

### 5.2. Use as a Food Additive 

The highly aromatic leaves of *P. amboinicus* are used in cooking to enhance the taste and aroma of food. The herb is often used as a substitute for oregano in the food trade. The leaves of *P. amboinicus* are added when marinating food, for food stuffings [104] and for flavoring meat dishes, e.g., beef, lamb and chicken [105,106]. The strong oregano-like flavor of this herb is an excellent choice to disguise the smell of meat while enhancing the taste. It is also used for the same reason in seafood cuisines such as fish and shellfish [67] to mask the smell of fish, which may prevent someone from enjoying their meal. In addition to this, *P. amboinicus* is used to spice dishes containing tomato sauces [10]. In India, the leaves are sometimes eaten raw with bread and butter or added to fritters. The tender and soft leaves are crunchy and have an astringent taste when chewed raw. They also may be added to beer and wine for flavoring [67]. 

## 6. Ornamental and Other Social Uses

*P. amboinicus* is grown as an ornamental in home gardens and hanging baskets for its attractive heart-shaped foliage and expression of fresh aroma when touched [10]. The variegated version with white-edged leaves, *Plectranthus amboinicus* “Variegata”, looks particularly attractive as an ornamental plant, especially when planted in hanging baskets or grown as a garden border. This plant is resistant to diseases and is able to survive drought due to its succulent leaves which can retain water. They can grow beautifully with minimum maintenance. Added to their culinary and medicinal value, *P. amboinicus* makes a preferred potted plant in many home gardens. *P. amboinicus* has scented leaves and these are often rubbed into the hair and body after bathing to replace body odor with a refreshing scent [67]. Many health and wellness spas around the world use essential oils of this plant to induce a calming sensation for aromatherapy. In the Amazon, the leaves are mixed with sugar and used as an intoxicant [68]. Whereas, in Tonga and Martinique, the leaves are used for the cleaning of textiles and to perfume them [10,46]. *P. amboinicus* is also used for spiritual and religious purposes and offered to the spirits when a house is being built in some community [67].

## 7. Conclusions and Recommendations

*P. amboinicus* is an important aromatic medicinal herb packed with many bioactive constituents and nutrients, which are important for maintaining good health. The plant has shown a wide range of biological properties and proved to be effective in curing respiratory, cardiovascular, oral, skin, digestive and urinary diseases. The biological properties are attributed to the occurrence of a wide range of bioactive compounds in the plant extracts as well as an essential oil. Thus, it can be stated that *P. amboinicus* has huge future prospects in meeting the global demand for natural, cost-effective and safer bioactive molecules in pharmaceutical and nutraceutical industries. However, additional research efforts are required to isolate, identify and interpret or authenticate the effectiveness of bioactive compounds from *P. amboinicus.* Though several classes of phytocompounds are isolated and authenticated from this herb, their bioactivity and toxicity studies under *in vivo* conditions using animal models are limited to only a few compounds. Till now, no scientific evidence is available on the human safety aspects of *P. amboinicus* even though it is used widely in folk medicine. Further, some detailed investigations should be aimed at understanding the effectiveness of these isolated compounds in treating other human illnesses.

## Figures and Tables

**Table 1:** Vernacular names and traditional uses of *Plectranthus amboinicus* commonly used by locals in their respective countries.

Country	Vernacular Names	Traditional Uses
Barbados	Poor man’s pork, Broad leaf thyme	Folk medicine, Culinary
Cambodia	Sak dam ray	Folk medicine, Culinary
China	Da shou xiang	Folk medicine, Home garden
Cuba	orégano; orégano de Cartagena	Folk medicine, Culinary
Fiji	Rhaivoki, Sage	Folk medicine, Culinary
Germany	Jamaika thymian	Folk medicine, Culinary
Guyana	Thick leaf thyme, broad leaf thyme	Folk medicine, Culinary
India	Indian Borage, Pashan Bhedi, Karpooravalli, Patharchur	Folk medicine, Culinary, Home garden
Indonesia	Torbangun, Daun Kutjing	Folk medicine, Culinary, Home garden
Malaysia	Daun bangun-bangun, Pokok bangun-bangun	Folk medicine, Culinary, Home garden
Philippines	Latai, Suganda, Oregano	Folk medicine, Culinary, Home garden
Puerto Rico	Puerto Rican oregano brujo, Cuban oregano	Folk medicine, Culinary
South Africa	Sup mint, French thyme, Indian mint	Folk medicine, Culinary, Home garden
Thailand	Hom duan huu suea, Niam huu suea	Folk medicine, Culinary
USA	Indian Borage, Country borage, Spanish thyme, Mexican mint, French thyme, Indian mint	Culinary, Home garden
Vietnam	Can day la	Folk medicine, Culinary
West Indies	French thyme, Spanish thyme, Broad-leaf thyme	Folk medicine, Culinary

**Table 2:** The known volatile constituents of *P. amboinicus.*

Compound Name	Formula	Plant Origin/Part	Method	References
Monoterpene hydrocarbons				
δ-3-Carene	C_10_H_16_	India, Malaysia, Morocco, Mauritius/Leaf	GC-MS	[29,30,31,32]
*p*-Cymene	C_10_H_14_	Brazil, India, Cambodia, Malaysia, Venezuela/Aerial parts, Leaf	GC-MS	[12,13,14,29,32,33,34,35,36,37,38]
Limonene	C_10_H_16_	India, Mauritius/Leaf	GC-MS	[29,30]
β-Myrcene	C_10_H_16_	Cambodia, India, Venezuela /Leaf	GC-MS	[13,29,34,36,38]
Ocimene	C_10_H_16_	Morocco/Leaf	GC-MS	[29,31]
α-Phellandrene	C_10_H_16_	India, Comoros, Mauritius, Venezuela/Leaf	GC-MS	[29,30,31,38]
β-Phellandrene	C_10_H_16_	India/Leaf	GC-MS	[14,35]
α-Pinene	C_10_H_16_	India, CambodiRa /Leaf	GC-MS	[29,36]
β-Pinene	C_10_H_16_	India/Leaf	GC-MS	[29]
Sabinene	C_10_H_16_	Cambodia,India, Morocco /Leaf	GC-MS	[29,31,36]
α-Terpinene	C_10_H_16_	India, Mauritius/Leaf	GC-MS	[14,29,30,34,35,39]
γ-Terpinene	C_10_H_16_	Brazil, Cambodia, Malaysia, Mauritius India, /Leaf	GC-MS	[13,14,29,30,32,34,35,36,37]
α-Terpinolene	C_10_H_16_	Morocco, Brazil/Leaf	GC-MS	[31,37]
α-Thujene	C_10_H_16_	India, Comoros, Venezuela/Leaf	GC-MS	[14,29,31,35,38]
Oxygenated monoterpenes				
Camphor	C_10_H_16_O	Comoros, Malaysia, Mauritius/Leaf	GC-MS	[30,31,32]
Carvacrol	C_10_H_14_O	Cambodia, India, Malaysia, Mauritius, Venezuela/Aerial parts, Leaf, Flower	GC-MS	[12,13,14,28,29,30,32,33,34,35,38,39,40,41,42,43]
Carvone	C_10_H_14_O	India/Leaf	GC-MS	[29]
1,8-Cineole	C_10_H_18_O	India/Leaf	GC-MS	[12,26,27,29,41]
Eugenol	C_10_H_12_O_2_	Cambodia, India/Leaf	GC-MS	[29,36,39,40,41,44]
Geraniol	C_10_H_18_O	Mauritius/Leaf	GC-MS	[30]
Linalool	C_10_H_18_O	Comoros, Mauritius /Leaf	GC-MS	[30,31]
Methyl carvacrol	C_11_H_16_O	India/Leaf	GC-MS	[29]
Methyl eugenol	C_11_H_14_O_2_	Cambodia/Leaf	GC-MS	[29,36]
α-Terpineol	C_10_H_18_O	India, Comoros, Venezuela /Leaf	GC-MS	[29,31,38]
Terpinen-4-ol	C_10_H_18_O	Brazil, India, Mauritius /Leaf	GC-MS	[12,13,29,30,31,37,39]
Thymol	C_10_H_14_O	Brazil, Cambodia, India, Venezuela/Aerial parts, Leaf	GC-MS	[12,13,14,29,33,34,35,37,38,39,40,41,44,45]
Thymol methyl ether	C_11_H_16_O	Brazil/Leaf	GC-MS	[37]
Sesquiterpene hydrocarbons				
α-Amorphene	C_15_H_24_	Cambodia/Leaf	GC-MS	[36]
Aromadendrene	C_15_H_24_	Brazil, India/Leaf	GC-MS	[34,37]
*trans*-α-Bergamotene	C_15_H_24_	Brazil, Comoros, India, Venezuela /Leaf, Aerial parts, Flower	GC-MS	[13,31,33,37,38]
*trans*-β-Bergamotene	C_15_H_24_	Cambodia/Leaf	GC-MS	[36]
γ-Cadinene	C_15_H_24_	Cambodia/Leaf	GC-MS	[36]
δ-Cadinene	C_15_H_24_	India, Cambodia/Leaf	GC-MS	[13,29]
α-Calacorene	C_15_H_20_	India/Aerial parts	GC-MS	[33]
*cis*-Calamenene	C_15_H_22_	Cambodia/Leaf	GC-MS	[36]
β-Caryophyllene	C_15_H_24_	Brazil, India, Venezuela /Leaf, Flower	GC-MS	[13,29,31,33,34,37,38,41]
γ-Caryophyllene	C_15_H_24_	India/Leaf	GC-MS	[42]
α-Copaene	C_15_H_24_	Comoros, India /Leaf	GC-MS	[29,31]
α-Cubebene	C_15_H_24_	India/Leaf, Aerial parts	GC-MS	[13,33]
(E,Z)-α-Farnesene	C_15_H_24_	France /Leaf	GC-MS	[29,46]
Germacrene D	C_15_H_24_	Cambodia/Leaf	GC-MS	[36]
γ-Gurjunene	C_15_H_24_	India/Aerial parts	GC-MS	[33]
Humulene	C_15_H_24_	Brazil, Cambodia, India, Morocco, Venezuela/Leaf, Aerial parts	GC-MS	[14,31,33,35,36,37,38,45]
α-Muurolene	C_15_H_24_	Cambodia, France, Mauritius/Leaf	GC-MS	[30,36,46]
Patchoulene	C_15_H_24_	India, Mauritius/Leaf	GC-MS	[30,42]
β-Selinene	C_15_H_24_	India, Comoros/Leaf	GC-MS	[14,31,35,44]
β-Sesquiphellandrene	C_15_H_24_	Cambodia/Leaf	GC-MS	[46]
Oxygenated sesquiterpenes				
Caryophyllene oxide	C_15_H_24_O	India, Cambodia, Venezuela /Leaf, Aerial parts	GC-MS	[13,14,29,33,35,36,38,39,44]
β-Cedrene epoxide	C_15_H_24_O	India/Aerial parts	GC-MS	[14,35]
β-Copaen-4-α-ol	C_15_H_24_O	India/Aerial parts	GC-MS	[14,35]
1-Epi-cubenol	C_15_H_26_O	India/Aerial parts	GC-MS	[14,35]
β-Eudesmol	C_15_H_26_O	India/Leaf	GC-MS	[29]
β-Himachalene oxide	C_15_H_24_O	India/Aerial parts	GC-MS	[14,35]
Humulene oxide	C_15_H_24_O	India/Leaf	GC-MS	[29]
Spathulenol	C_15_H_24_O	India/Leaf	GC-MS	[12,39]
Others (Terpenes, phenylpropanoids, esters, fatty acids, alcohols, aldehyde)				
1,2-Benzenediol 4-(1,1 dimethylethyl)	C_10_H_14_O_2_	India/Leaf	GC-MS	[44]
Chavicol	C_9_H_10_O	India/Leaf	GC-MS	[27,40]
Methyl chavicol	C_10_H_12_O	India/Aerial parts	GC-MS	[14,33,35]
α-Corocalene	C_15_H_20_	India/Aerial parts	GC-MS	[33]
Dihydro carveol	C_10_H_18_O	India/Aerial parts	GC-MS	[14,35]
Durohydroquinone	C_10_H_14_O_2_	India/Leaf	GC-MS	[44]
1,4 Eicosadiene	C_20_H_38_	India/Leaf	GC-MS	[44]
Ethyl Salicylate	C_9_H_10_O_3_	India/Leaf	GC-MS	[27,40]
(Z)-1,3-Hexadiene	C_6_H_10_	France /Leaf	GC-MS	[46]
(Z)-3-Hexen-1-ol	C_6_H_12_O	France/Leaf	GC-MS	[29,46]
Methyl octanoate	C_9_H_18_O_2_	India/Aerial parts	GC-MS	[14,35]
1-Octen-3-ol	C_8_H_16_O	India, Mauritius, Venezuela/Leaf	GC-MS	[29,30,38,39]
Oleic acid	C_18_H_34_O_2_	India/Leaf	GC-MS	[44]
2-Phenyl ethyl tiglate	C_13_H_16_O_2_	India/Aerial parts	GC-MS	[14,35]
Phytol	C_20_H_40_O	India/Leaf	GC-MS	[44]
Squalene	C_30_H_50_	India/Leaf	GC-MS	[44]
Tetradecanal	C_14_H_28_O	India/Aerial parts	GC-MS	[14,35]
3,7,11,15–Tetramethyl-2-hexadecen-1-ol	C_20_H_40_O	India/Leaf	GC-MS	[44]
Thymol acetate	C_12_H_16_O_2_	India/Leaf	GC-MS	[29,39]
Trans-sabinene hydrate	C_12_H_20_O_2_	India/Aerial parts	GC-MS	[14,35]
Undecanal	C_11_H_22_O	India/Aerial parts	GC-MS	[14,35]

**Table 3:** The known non-volatile constituents of *P. amboinicus.*

Compound Name	Formula	Plant Origin/Part	Analytical Method	References
Phenolic acids				
Caffeic acid	C_9_H_8_O_4_	India, Egypt/Leaf, stem, root (Methanol extract)	UV/NMR/UPLC/MS/HPLC	[48,51]
Gallic acid	C_7_H_6_O_5_	India/Stem (Methanol extract)	HPLC	[51]
*p*-Coumaric acid	C_9_H_8_O_3_	India, Egypt/Leaf, stem, root (Methanol and ethyl acetate fraction)	UV/NMR/UPLC/MS/HPLC	[48,51]
Rosmarinic acid	C_18_H_16_O_8_	India, Egypt, Thailand/Leaf, stem, root (Methanol and ethyl acetate fraction)	UV/NMR/UPLC/MS/HPLC	[48,51,52]
Salvianolic acid A	C_26_H_22_O_10_	Thailand/Aerial parts (Water extract)	UV/NMR/MS/HPLC	[52]
Shimobashiric acid	C_36_H_32_O_16_	Thailand/Aerial parts (Water extract)	UV/NMR/MS/HPLC	[52]
Flavonoids				
Chrysoeriol	C_16_H_12_O_6_	Philippines, Egypt/Leaf, stem, root (Chloroform extract; Ethyl acetate fraction)	UV/NMR/UPLC/MS	[48,49]
Cirsimaritin	C_17_H_14_O_6_	Philippines/Leaf (Chloroform extract)	UV/NMR	[49]
Eriodictyol	C_15_H_12_O_6_	Egypt/Leaf, stem, root (Ethyl acetate fraction)	UV/NMR/UPLC/MS	[48]
Luteolin	C_15_H_10_O_6_	Egypt/Leaf, stem, root (Ethyl acetate fraction)	UV/NMR/UPLC-MS	[48]
Rutin	C_27_H_30_O_16_	India/Stem (Methanol extract)	HPLC	[51]
Salvigenin	C_18_H_16_O_6_	Philippines/Leaf (Chloroform extract)	UV/NMR	[49]
Thymoquinone	C_10_H_12_O_2_	Thailand/Aerial parts (Water extract)	UV/NMR/MS/HPLC	[52]
Quercetin	C_15_H_10_O_7_	Egypt/Leaf, stem, root (Ethyl acetate fraction)	UV/NMR/UPLC/MS/HPLC	[48,51]
5,4′-Dihydroxy-6,7-dimethoxy flavone	C_17_H_14_O_6_	Egypt/Leaf, stem, root (Ethyl acetate fractions)	UV/NMR/UPLC/MS	[48]
5,4′-Dihydroxy-3,7-dimethoxy flavone	C_17_H_14_O_6_	Egypt/Leaf, stem, root (Ethyl acetate fractions)	UV/NMR/UPLC/MS	[48]
5-*O*-Methyl-luteolin	C_16_H_12_*O*_6_	Egypt/Leaf, stem, root (Ethyl acetate fractions)	UV/NMR/UPLC/MS	[48]
3,5,7,3′,4′-Pentahydroxy flavanone	C_15_H_12_*O*_7_	Egypt/Leaf, stem, root (Ethyl acetate fractions)	UV/NMR/UPLC/MS	[48]
4′,5,7-Trihydroxyflavone (apigenin)	C_15_H_10_O_5_	Egypt/Leaf, stem, root (Ethyl acetate fractions)	UV/NMR/UPLC/MS	[48]

**Table 4:** Pharmacological properties of *P. amboinicus* different parts.

Pharmacological Activity	Plant Part Used	Bioactive Compound	Potential Effect	References
Antibacterial activity	Leaf extract/Essential oil/Decoction	Biogenic zinc oxide nanoparticles	Pam-ZnO NPs control the growth of methicillin-resistant *Staphylococcus aureus* biofilm; inhibits growth *of Escherichia coli*, *Salmonella typhimurium* & *Mycobacterium tuberculosis.*	[54,55,56,57,58,59,60,61,62,63]
Antifungal activity	Leaf extract/Essential oil	Carvacrol, *p-*Cymene, α-Terpinolene & β-caryophyllene	Fungitoxic properties against *Aspergillus flavus*, *Aspergillus niger, Aspergillus ochraceus, Aspergillus oryzae*, *Candida versatilis, Fusarium* sp. *GF-1019, Penicillium* sp., *Saccharomyces cerevisiae, Candida albicans*, *C. tropicalis*, *C. krusei* & *C. stellatoidea.*	[13,63]
Antiviral activity	Leaf/Ethanolic extract	-	Exhibited antiviral activity against viruses (*VSV, HSV1* & *HIV*)*.*	[28,64,65,66]
Activity against Respiratory diseases	Leaf extract/Decoction or juice/Essential oil	-	Used as folk medicine in brazil for influenza, cough, expectorant, bronchitis and throat problems; given orally to control asthma & catarrh; used as bronchodilator.	[11,12,67,68,69,70,71,72]
Lavicidal potential	Leaf extract/Essential oil	Pam-ZnO NPs (zinc oxide nanoparticles)	Exhibited up to 100% mortality in *Anopheles stephensi, Culex quinquefasciatus* & *Culex tritaeniorhynchus*.	[14,47,62,73,74,75]
Oral Diseases	Essential oil	Carvacrol	Antagonistic effect when used with mouthwash.	[76]
Digestive diseases (Diarrhea, Constipation, dyspepsia, indigestion & as carminative)	Leaf extract/Juice	-	Stimulates growth of *Lactobacillus plantarum* and inhibits growth of selected food-borne pathogens (*Escherichia coli* & *Salmonella typhimurium)*; relieves constipation troubles; prevents formation of gas in the gastrointestinal tract & facilitates expulsion of gas	[30,56,67,68,77]
Antitumor activity	Leaf extract/Crude hydro alcoholic extracts	Flavone (Luteolin), flavonols	Inhibited the growth of sarcoma 180 & Ehrlich ascite carcinoma tumors in mice; showed significant anticancer activity through inducing apoptosis in A549 (human lung cancer) cell line.	[78,79]
Antiinflammatory activity	Aerial part/Ethanol, methanol & hexane extract	Rosmarinic acid, Shimobashiric acid, alvianolic acid L, Rutin, Thymoquinone, Quercetin	Concentration of 0.1 mg/mL inhibited 10%–50% DNA binding activities; inhibited the binding of AP-1 to its consensus DNA sequence; decreased carrageenan-induced paw edema up to 40%; significantly increased IgG, IgM & lysozyme activity in rats.	[34,50,52,78,80]
Analgesic activity	Leaf extract	-	Provides remedy for headache, backache & musculo-skeletal problems.	[52,81,82,83]
Wound healing activities	Leaf & Root Aqueous extract	-	Increased wound healing activity in experimentally induced diabetic mice & againt murrels.	[60,84,85,86,87]
Cardiovascular disorders	Leaf aqueous extract	-	Positive inotropic activity in the isolated frog heart; effective for treating congestive heart failure.	[67,88]
Skin disease (Anti-dandruff, Cuts, Skin Allergy; Burns)	Leaf extract/Essential oil/Leaf juice/Paste	Thymol, 1,8-Cineole, β-Pinene, α-pinene, phenolic compounds	Inhibits the growth of *Malassezia furfur*; applied on cut as antiseptic promoted better healing; paste was effective against skin allergies, skin burns.	[68,89,90,91,92]
Insect bites	Leaf aqueous extracts	-	Potency as antidote for scorpion *(Heterometrus laoticus)* venom with >50% efficiency.	[68,93]
Lactogenic properties	Leaf	Nutrient content (iron & carotene)	Increased breast milk in new mothers.	[80]
Anti-epileptic activity	Leaf, stem, root Extract (aqueous & alcoholic)	Alkaloids, flavonoids & saponins	Effective as an anticonvulsive and/or antiepileptic medicine.	[11,68,94]
Activity against Genitourinary diseases	Leaf Decoction/Ethanolic & aqueous leaf extract	-	Effective against urinary diseases in the Amazon & India; to relieve kidney troubles, treat vaginal discharges; used after childbirth; increased urine volume & electrolyte concentration in male albino rats.	[67,69,95,96,97,98]
Antioxidant activity	Leaf extracts/Essential oil	Carvocrol & Thymol	Exhibited significant inhibition in DPPH free radical & hydroxyl radical formation.	[16,34,99,100]
Other diseases	Leaf	-	Fevers, meningitis, eye diseases.	[67,101,102,103]

**Table 5:** Nutritional content of *P. amboinicus.*

No.	Principles	Nutrient Content
1.	Proteins	0.6%
2.	Vitamins	
**	*+ Ascorbic acid*	0.003%
**	*+ Thiamine*	0.00008%
3.	Minerals	
**	*+ Calcium*	0.158%
**	*+ Phosphorous*	0.016%
**	*+ Potassium*	0.138%
**	*+ Sodium*	0.0047%
**	*+ Magnesium*	0.088%
4.	Trace metals	
**	*+ Iron*	0.262%
**	*+ Zinc*	0.0003%
**	*+ Copper*	0.00012%
**	*+ Chromium*	0.000022%
5.	Soluble dietary fibers	0.31%
6.	Insoluble dietary fibers	1.56%
7.	Phytic acid	0.00092%
8.	Soluble oxalate	0.02%

## Abbreviations

The following abbreviations are used in this manuscript:

SPME: Solid phase micro extraction
GC: Gas chromatography
LSC: Liquid-solid chromatography
GLC: Gas-liquid chromatography
GC-MS: Gas chromatography-mass spectroscopy
GC-FID: Gas chromatography outfitted with flame ionization detector (GC-FID)
NMR: Nuclear magnetic resonance
UV: Ultra violet
HPLC: High pressure liquid chromatography
UPLC: Ultra performance liquid chromatography
GAE: gallic acid equivalent
TAE: Tannic acid equivalent
RE: Rutin equivalent
Pam-ZnO NPs: P. amboinicus Zinc oxide nanoparticles
TNF-α: Tumor necrosis factor
COX-2: Cyclooxygenase 2
AP-1: Activator protein-1
HWE: Hot water extract (HWE)
HE: Hexane extract
SDS-PAGE: Sodium dodecyl sulfate polyacrylamide gel electrophoresis
HSV 1: Herpes simplex virus-1
STDs: Sexually-transmitted diseases
AIDS: Acquired immune deficiency syndrome (AIDS)
HIV: Human immunodeficiency virus
VSV: Vesicular stomatitis virus
